# Sensory experiences and cues among E-cigarette users

**DOI:** 10.1186/s12954-020-00420-0

**Published:** 2020-10-15

**Authors:** J. DiPiazza, P. Caponnetto, G. Askin, P. Christos, M. Lyc Psych Maglia, R. Gautam, S. Roche, R. Polosa

**Affiliations:** 1 Hunter Bellevue School of Nursing, Hunter College-City University of New York, New York, NY USA; 2 Clinical and Translational Science Center, Weill Cornell Medicine, New York, NY USA; 3 Centro per la Prevenzione e Cura del Tabagismo (CPCT), Azienda Ospedaliero-Universitaria “V.Emanuele-Policlinico”, Università di Catania, Catania, Italy; 4 Institute of Internal Medicine, Azienda Ospedaliero-Universitaria “Policlinico V. Emanuele”, Università di Catania, Catania, Italy; 5 Institute for Social Marketing, University of Stirling, Stirling, FK9 4LA UK; 6 Division of Biostatistics and Epidemiology, Department of Healthcare Policy and Research, Weill Cornell Medicine, New York, NY USA; 7 Center of Excellence for the Acceleration of Harm Reduction (CoEHAR), University of Catania, Catania, Italy; 8 Department of Clinical and Experimental Medicine, University of Catania, Catania, Italy

**Keywords:** Smoking cessation, Smoking, Electronic cigarettes, Sensory, Smoking cues, Nicotine, Tobacco

## Abstract

**Background and aims:**

We characterized the extent and quality of respiratory sensations and sensory-related smoking cues associated with e-cigarette use among those who failed to quit combustible tobacco cigarette (CTC) use with traditional FDA approved medications but succeeded in doing so with e-cigarettes. Further, we sought to understand former smokers’ perceptions about the influence of sensory experience with e-cigarette use on CTC cessation outcomes.

**Methods:**

A nonrandom purposive sample of 156 participants recruited in the USA through the Consumer Advocates for Smoke Free Alternatives Association Facebook page completed an online cross-sectional survey to assess sensory experiences and smoking cues associated with e-cigarette use. Descriptive statistics were calculated, and the ANOVA/Kruskal–Wallis test with post hoc testing and the two-sample *t *test/Wilcoxon rank-sum test, as appropriate based on distribution, were used to assess the association between sample characteristics and sensory experiences and cues using investigator constructed questions, the Modified Cigarette Evaluation Questionnaire (mCEQ) and the Smoking Cue Appeal Survey (SCAS).

**Results:**

With e-cigarette use, participants reported feeling the vapor in their throats, windpipes, noses, lungs, and on their tongues; reductions in nicotine craving; and enjoyment of their e-cigarette, including tasting, smelling, and seeing the vapor and touching the device. Women had greater craving reduction than men (*p* = 0.023). Those who began smoking at 13 years of age or younger had more satisfaction and had greater sensory enjoyment than those who began smoking at 16–17 years of age (*p* = 0.015 and *p* = 0.026, respectively), as well as greater sensory enjoyment than those who began smoking at 14–15 years of age (*p* = 0.047). There was a significant overall association between the number of years a respondent smoked and e-cigarette sensory enjoyment (*p* = 0.038). Participants 18–34 years old rated e-cigarettes as being more pleasant compared to 45 + years olds, (*p* = 0.012). Eighty-four percent of participants reported the sensation of the vapor as important in quitting CTCs, and 91% believed the sensations accompanying e-cigarette use contributed to their smoking cessation success.

**Conclusions:**

For those who failed to quit previously using approved cessation medications to stop smoking cigarettes, sensory experiences associated with e-cigarette use may help smokers quit smoking.

## Background

Smoking is reinforced by a variety of sensory experiences [1], but none of the US FDA approved smoking cessation medications [2] are specifically designed to address the “sensory impact” [3] smokers report as desirable, satisfying, and reinforcing to their smoking behavior, such as throat scratch, heat or coolness in the upper and lower airways, flavor [4, 5], and various sensations on the tongue, nose, throat, windpipe, and chest [3, 6, 7]. Examining sensory impact as an influencer of cessation outcomes is critical because, while outcomes improve with use of approved cessation medications as compared to no treatment, relapse rates among treated smokers are still estimated to be as high as 50% one year post quit [8–12]. Indeed, cessation outcomes following the use of approved cessation medications are modest when considered in the context of the extent of morbidity and early mortality resulting from smoking. Worldwide tobacco use causes an estimated six million deaths annually [13], including an estimated 480,000 in the United States [14].

It is concerning and perplexing that none of the US-approved cessation medications address the sensory impacts of smoking. Today, it is well understood that in addition to nicotine, CTC smoking is reinforced by a variety of sensory experiences [1], suggesting the need to address multiple influences in smoking cessation efforts. In fact, research suggesting the pairing of nicotine replacement and sensory reinforces for CTC cessation dates as far back as the seminal work of Cain, Rose and colleagues [15, 16]. In 1988, the “airway sensory hypothesis” (ASH) was proposed [17], whose foundation built upon research [18, 19] influenced by Cain’s [15], seminal work concerning the sensory attributes of cigarette smoking. In addressing smoking addiction beyond the physical addiction to nicotine, ASH suggests that research efforts regarding CTC cessation should be directed toward ways of manipulating airway sensations to influence smoking behavior and improve cessation outcomes [17].

In fact, the tobacco industry’s (TI’s) approach to ingrain smoking is to both maximize the physical addiction to nicotine and the sensory appeal of smoking. The TI has extensive programs of sensory focused research, spanning decades and supported by large budgets [20, 21]. In their laboratories, TI scientists developed a keen understanding of how to alter proportions of cigarette ingredients to create distinct sensory responses when smoked. To further entrench smoking, cigarettes are custom-designed through extensive marketing research to attract specific populations by delivering distinct sensory features [20]. For example, cigarettes were custom designed for women over age 35 who prefer a milder and less harsh feel in the respiratory tract and black men who prefer stronger airway sensations [20].

The emergence of e-cigarettes, battery-operated devices that vaporize a solution consisting of glycerol, propylene glycol, distilled water, and flavorings, which may or may not contain nicotine, [22] has the potential to address the sensory impact of CTC as a cessation intervention. In fact, research focused on the sensory aspects of e-cigarette use is developing [23–26]. The e-cigarette user inhales the aerosol, referred to as “vaping,” with e-cigarettes sharing many similarities with smoking in the behavioral aspect of their use [27]. Many users of e-cigarettes are current or former smokers who report using them long-term as an alternative to CTCs, to reduce cigarette consumption or quit smoking [28, 29], to relieve tobacco withdrawal symptoms [30], and/or to continue having a “smoking experience without smoking” [31, 32].

The goal of this study was to characterize the extent and quality of respiratory sensations and sensory-related smoking cues associated with e-cigarettes. Study participants previously tried non-sensory stimulating approved cessation medications to quit combustible tobacco cigarette (CTC) without success but were maintaining cessation with a sensory stimulating e-cigarette. Further, we examined the value participants placed on the sensory aspects of e-cigarette use to influence cessation outcomes. To our knowledge, there are no other studies that have studied our unique population.

In this study, we sought to characterize the extent and quality of respiratory sensations and sensory-related smoking cues associated with e-cigarette use among a group of former CTC smokers. We also examined the value participants placed on the sensory aspects of e-cigarette use to influence cessation outcomes. These individuals previously failed to quit CTC smoking using non-sensory stimulating approved cessation medications, but successfully quit with e-cigarettes, suggesting the potential of e-cigarettes or other interventions that address sensory aspects of smoking to serve as a CTC cessation intervention.

## Methods

### Participants and recruitment

Through the posting of an advertisement on the Consumer Advocates for Smoke Free Alternatives Association (CASAA) Facebook page at https://www.facebook.com/CASAAmedia, a convenience sample (*N* = 156) of adult e-cigarette users was recruited to complete an anonymous cross-sectional Web-based survey. Clicking a Web-link from the Facebook page allowed potential participants to learn about the study with an option to move forward to screening, consenting, and answering the survey. Demographic and smoking history questionnaires were all to be completed within the Qualtrics platform, an online data collection tool (Qualtrics, LLC). Informed consent was obtained from all individual participants included in the study.

Eligible participants had quit smoking CTCs for at least the last 3 months, were currently using e-cigarettes and no other intervention as a smoking cessation aide, and had at least one unsuccessful attempt at smoking cessation using one or more first-line approved cessation medications: Varenicline, Buproprion, or nicotine gum, inhaler, lozenge, nasal spray, or patch. A $10 electronic gift card was offered to participants who completed the survey [33].

### Measures

The survey included investigator-developed questions that elaborated on the presence and quality of sensory experiences and cues associated with e-cigarette use and the importance of sensory experiences’ influence on cessation outcomes. The survey also included the Modified Cigarette Evaluation Questionnaire (mCEQ) [34]. Individuals responded to each of the mCEQ questions on a Likert scale, with 1 = not at all to 7 = extremely. Five domain scores were created by summing the appropriate domain-specific items based on prior confirmatory factor analysis [34]. The five domains of the 11-item mCEQ included Smoking Satisfaction (adapted to Vaping Satisfaction), Psychological Reward, Enjoyment of Respiratory Tract Sensation, Craving Reduction, and Aversion.

In addition, the survey included the Smoking Cue Appeal Survey (SCAS), a measure of the sensory cue appeal of smoking [35] for which reliability and validity have also been established. Each item is rated using a Likert-scale ranging from 3 (extremely unpleasant) to − 3 (extremely pleasant) assessing the sight, smell, taste, and tactile sensation of e-cigarette use [35]. The SCAS total score was calculated by summing all item scores.

We note that the mCEQ and the SCAS have been primarily used to evaluate respiratory sensations and sensory cues of CTC smokers, but were used here to examine those related to e-cigarette use. Therefore, the measures were adapted to refer to e-cigarettes and e-cigarette use. Demographic and smoking history questions were used to describe the sample. All data were de-identified and collected on July 13, 2017.

### Statistical analysis

Descriptive statistics (i.e., mean, standard deviation [SD], median, interquartile range [IQR], frequency, and/or percent) were calculated for patient demographics, smoking history and responses to the investigator-developed questions. Descriptive statistics were also calculated for the 5 domains of the mCEQ, and for the SCAS questions in which responses of “Pleasant,” “Very Pleasant,” and “Extremely Pleasant” was collapsed into a single category. Continuous variables were described as mean ± SD, or median [25th percentile; 75th percentile]. Discrete variables were described as frequencies (%).

Analysis of variance (ANOVA) or the Kruskal–Wallis test, as appropriate based on distribution, was used to assess the associations between categorical variables of interest and (1) the 5 mCEQ domains and (2) the Total SCAS score. Categorical variables of interest included baseline age category (18–34, 35–44, 45+), residence type (rural, suburban or urban), age range when the participant began smoking (≤ 13 years, 14–15 years, 16–17 years, 18+ years), number of years smoked (≤ 15 years, 16–25 years, 26–35 years, 36+ years), and number of times attempted quitting (< 5 times, 6–10 times, 11+ times). When overall differences were observed, post-hoc tests (Tukey HSD and Dunn–Holm) were used to assess differences between pairs of groups and adjust for multiple comparisons. The two-sample *t* test or Wilcoxon rank-sum test, as appropriate, was used to assess the associations between binary variables of interest (gender and daily number of cigarettes smoked (≤ 20, 21+) and mCEQ domains and the SCAS Total score. The chi-square test or Fisher’s exact test, as appropriate based on expected cell counts, was used to assess the association between discrete variables of interest and the above outcomes. All *p* values were two-sided with statistical significance evaluated at the 0.05 alpha level. Ninety-five percent confidence intervals (95% CIs) were computed to assess the precision of the obtained estimates. All analyses were performed using R Version 3.3.1 (R Foundation for Statistical Computing, Vienna, Austria).

## Results

### Participant Characteristics and smoking history

Table 1 describes participants’ sociodemographic and smoking history characteristics.

**Table 1:** Participant characteristics and smoking history

Demographics	*n* (%)
Gender	
Female	85 (55.6%)
Male	58 (44.4%)
Age (in years)	
18–24	2 (1.30%)
25–34	49 (31.8%)
35–44	36 (23.4%)
45–54	57 (37.0%)
55–64	10 (6.49%)
Residence	
Urban	35 (22.9%)
Suburban	66 (43.1%)
Rural	52 (34.0%)
Race	
White	
Yes	152(97.4%)
No	4 (2.6%)
African American	
Yes	2(1.3%)
No	154 (98.7%)
Asian American	
Yes	2(1.3%)
No	154 (98.7%)
American Indian	
Yes	10 (6.4%)
No	146 (93.6%)
Smoking history *n* (%)	
Age started smoking	
≤ 13 years	33 (21.4%)
14–15 years	53 (34.4%)
16–17 years	43 (27.9%)
≥ 18 years	25 (16.2%)
Years smoked	
≤ 15 years	46 (30.9%)
16–25 years	37 (24.8%)
26–35 years	40 (26.8%)
≥ 36 years	26 (17.4%)
Cigarettes per day	
≤ 20	72 (47.4%)
≥ 21	80 (52.6%)
Number of previous quit attempts	
≤ 5 times	75 (54.7%)
6–10 times	34 (24.8%)
≥ 11 times	28 (20.4%)

Smokers typically make multiple quit attempts [36] as was true of almost half of our participants. The majority of participants began smoking before age 18, smoked for at least 16 years, and about half reported smoking more than 20 cigarettes per day. The vast majority of the sample identified as “White” with those aged 45–54 the most represented. There were slightly more females and most lived in suburbia or rural areas.

### Investigator-developed questions

#### Presence and quality of sensory experiences and cues using an e-cigarette

Responses from ten investigator-developed questions indicated that the majority of participants experienced respiratory sensations when using their e-cigarettes and enjoyed the sensations (Table 2). The presence of sensations varied based on their location in the respiratory tract, with sensations in the throat being the most common and in the lungs the least. Enjoyment of sensations varied, with the tongue and throat being the most enjoyable.

**Table 2:** Respiratory tract sensations and enjoyment reported during e-cigarette use

Sensation^a^	Experiences the sensation (%)	95% CI	Enjoys the sensation^b^ (%)	95% CI
Throat	97	93%, 99%	95	90%, 98%
Tongue	94	89%, 97%	98	94%, 99%
Windpipe	75	67%, 81%	86	80%, 91%
Nose	72	64%, 79%	85	78%, 90%
Lungs	67	60%, 74%	82	75%, 87%

^a^Sensation: Investigator-developed questions: I feel the vapor in my throat when I use my e-cigarette; I feel the vapor on my tongue when I use my e-cigarette; I feel the vapor in my windpipe when I use my e-cigarette; I feel the vapor in my nose when I use my e-cigarette; I feel the vapor in my lungs when I use my e-cigarette

^b^Enjoys the Sensation: Investigator-developed questions: I enjoy the feeling of the vapor in my throat when I use my e-cigarette; I enjoy the feeling of the vapor on my tongue when I use my e-cigarette; I enjoy the feeling of the vapor in my windpipe when I use my e-cigarette; I enjoy the feeling of the vapor in my nose when I use my e-cigarette; I enjoy the feeling of the vapor in my lungs when I use my e-cigarette.

#### Importance of sensory experiences’ influence on cessation outcomes

Table 3 describes participants’ responses to the importance of sensations and the extent to which they believed these sensations contributed to their ability to quit smoking. Participants were asked to rate on a 7-point Likert scale (Not at all (1) to extremely (7) the importance of the sensation of the vapor. The proportion of responses to the statement, “The feeling of the vapor when I used my e-cigarette is important to me,” was: not at all (5.03%), very little (5.66%), a little (5.03%), moderately (17.61%), a lot (18.87%), quite a lot (25.16%), and extremely (22.64%). Together, eighty-four percent of participants reported the sensation of the vapor as moderately to extremely important.

**Table 3:** Importance of sensory experiences’ influence on cessation outcomes

Statement	Not at all (1) (%)	Very little (2) (%)	A little (3) (%)	Moderately (4) (%)	A lot (5) (%)	Quite A lot (6) (%)	Extremely (7) (%)
The feeling the vapor is important to me when I use my e-cigarette	5.03	5.66	5.03	17.61	18.87	25.16	22.64
The feeling of the vapor when I use my e-cigarette contributed to my smoking cessation success	3.18	1.91	3.82	7.01	11.46	20.38	52.23

Participants were asked to rate on a 7-point Likert scale (Not at all (1) to extremely (7)) the extent to which sensations associated with e-cigarette use influenced cessation outcomes. The proportions of responses to the statement, “The feeling of the vapor when I use my e-cigarette contributed to my smoking cessation success,” were: not at all (3.18%), very little (1.91%), a little (3.82%), moderately, (7.01%), a lot (11.46%), quite a lot (20.38%), and extremely (52.23%). Together, ninety one percent of participants believed the sensations accompanying e-cigarette use contributed moderately to extremely to their smoking cessation success.

### The Modified Cigarette Evaluation Questionnaire (mCEQ)

As can be seen in Table 4, mean scores on the five domains of the mCEQ based on the 7 point Likert scale 1 (not at all) to 7 (extremely) indicate that participants reported high levels of satisfaction (mean of 19.7 out of a maximum of 21), enjoyment (mean of 5.6 out of a maximum of 7), and craving reductions (mean of 6.0 out of a maximum of 7); moderate levels of psychological reward (mean of 22.2 out of a maximum of 35); and low levels of aversion to e-cigarette use (mean of 2.4 out of a maximum of 14). In fact, ninety percent of participants reported no aversion to e-cigarette use with questions specifically asking about nausea and dizziness.

**Table 4:** Mean and median scores on the five domains of the mCEQ

Domain^a^	Mean (SD) score	Median [IQR] score	*N*
Vaping satisfaction	19.7 (2.0)	21.0 [19.0; 21.0]	155
Psychological reward	22.2 (6.3)	22.0 [18.0; 26.5]	155
Enjoyment of respiratory tract sensations	5.6 (1.4)	6.0 [5.0; 7.0]	156
Craving reduction	6.0 (1.3)	6.0 [6.0; 7.0]	156
Aversion	2.4 (1.2)	2.0 [2.0; 2.0]	155

^a^Smoking Satisfaction = average of mCEQ questions #1 (Was e-cigarette use satisfying?), #2 (Does the e-cigarettes taste good?), and #12 (Do you enjoy using your e-cigarette?); Psychological Reward = average of mCEQ questions #4 (Does using your e-cigarette calm you down?), #5 (Does using your e-cigarette make you feel more awake?), #6 (Does using your e-cigarette make you feel less irritable?), #7 (Does using your e-cigarette help you concentrate?), and #8 (Does using your e-cigarette reduce your hunger for food?); Enjoyment of Respiratory Tract Sensation: mCEQ questions #3 (Do you enjoy the sensation in your throat and chest?); Craving Reduction: mCEQ question #11 (Does using your e-cigarette immediately reduce your craving for nicotine?); and Aversion = average of mCEQ questions #9 (Does using your e-cigarette make you dizzy?) and #10 (Does using your e-cigarette make you nauseous?). Questions on the mCEQ were adapted to refer to e-cigarettes and e-cigarette use. Vaping Satisfaction = average of mCEQ questions #1 (Was e-cigarette use satisfying?), #2 (Does the e-cigarettes taste good?), and #12 (Do you enjoy using your e-cigarette?); Psychological Reward = average of mCEQ questions #4 (Does using your e-cigarette calm you down?), #5 (Does using your e-cigarette make you feel more awake?), #6 (Does using your e-cigarette make you feel less irritable?), #7 (Does using your e-cigarette help you concentrate?), and #8 (Does using your e-cigarette reduce your hunger for food?); Enjoyment of Respiratory Tract Sensation: mCEQ questions #3 (Do you enjoy the sensation in your throat and chest?); Craving Reduction: mCEQ question #11 (Does using your e-cigarette immediately reduce your craving for nicotine?); and Aversion = average of mCEQ questions #9 (Does using your e-cigarette make you dizzy?) and #10 (Does using your e-cigarette make you nauseous?). Questions on the mCEQ were adapted to refer to e-cigarette use. Results were adapted to vaping rather than smoking.

As can be seen in Table 5, scores on the vaping satisfaction and enjoyment domains of the mCEQ varied significantly according to the age at which the participant began smoking (13 years or younger, 14–15 years, 16–17 years or 18 years or older). ANOVA yielded differences between the four age groups on the mCEQ Satisfaction domain (overall *p* = 0.013), and the post hoc Tukey HSD test indicated that the 16–17-year-old group (mean ± sd: 19.2 ± 1.97) had a significantly lower mCEQ Satisfaction score compared to the 13-year-old and younger group (mean ± sd: 20.4 ± 1.23), *p* = 0.015. The non-parametric Kruskal–Wallis test indicated an overall association between the age the participant began smoking and the mCEQ Enjoyment domain score (overall *p* = 0.017). Post hoc testing (Dunn–Holm) showed a significant difference in the median mCEQ Enjoyment score, between the 16–17-year-old group (Median [IQR] of 5.00 [4.00;6.00]) and both the 14–15-year-old group (Median [IQR] of 6.00 [5.00;7.00]) and the 13-year-old and younger group (Median [IQR] of 6.00 [5.00;7.00]), *p* = 0.026 and *p* = 0.047, respectively. In addition, enjoyment of e-cigarette use differed according to years smoked (overall *p* = 0.038). After adjusting for multiple comparisons, there was a trend observed between those who smoked 26–35 years versus 36 or more years, with a p value of 0.061 for the pairwise comparison, suggesting that the test might be underpowered to detect the difference.

**Table 5:** Association of demographics and smoking history and domains of the modified cigarette evaluation questionnaire (mCEQ)

mCEQ domains^a^	Vaping satisfaction	Psychological reward	Enjoyment of respiratory tract sensations	Craving reduction	Aversion
Gender					
Male	19.5 ± 1.94	22 (19.0; 25.2)	6.0 (5.0; 7.0)	6.0 (5.0; 7.0)	2.0 (2.0; 2.0)
Female	19.8 ± 2.15	23 (18.0; 28)	6.0 (5.0; 7.0)	7.0 (6.0; 7.0)*	2.0 (2.0; 2.0)
Baseline age					
18–34	19.7 ± 2.01	23.0 (21.0; 26.5)	6.0 (5.0; 7.0)	7.0 (6.0; 7.0)	2.0 (2.0; 2.0)
35–44	20.2 ± 1.33	21.0 (15.526.0)	6.0 (4.0; 7.0)	6.5 (5.75; 7.0)	2.0 (2.0; 2.0)
45+	19.5+/2.29	23.0 (18.0; 27.0)	6.0 (5.0; 7.0)	6.0 (5.0; 7.0)	2.0 (2.0; 2.0)
Residence type					
Rural	19.9 ± 1.93	22.5 (19.0; 26.0)	6.0 (5.0; 7.0)	6.0 (6.0; 7.0)	2.0 (2.0; 2.0)
Suburban	19.7 ± 1.74	22.0 (17.2; 26.8)	6.0 (4.25; 7.0)	7.0 (5.25; 7.0)	2.0 (2.0; 2.0)
Urban	19.3 ± 2.73	23.0 (19.0; 27.5)	6.0 (5.0; 7.0)	7.0 (4.50; 7.0)	2.0 (2.0; 2.0)
Age began smoking					
< 13 years	20.4 ± 1.23*	23.0 (20.0; 27.0)	6.0 (5.0; 7.0)**	7.0 (6.0; 7.0)	2.0 (2.0; 2.0)
14–15 years	*20.1 ± 1.26	22.0 (18.0; 27.0)	6.0 (5.0; 7.0)	7.0 (6.0; 7.0)	2.0 (2.0; 2.0)
16–17 years	19.2+/1.97**	22.0 (18.0; 26.0)	5.0 (4.0; 6.0)**	6.0 (6.0; 7.0)	2.0 (2.0; 2.0)
18+ years	19.5 ± 2.52	24.0 (20.0; 26.0)	5.0 (5.0; 7.0)	6.0 (5.0; 7.0)	2.0 (2.0; 2.0)
Years smoked					
≤ 15 years	19.6 ± 2.06	23.0 (21.0; 27.8)	6.0 (5.0; 7.0) *	6.0 (6.0; 7.0)	2.0 (2.0; 2.0)
16–25 years	19.9 ± 1.82	22.0 (17.0; 26.0)	6.0 (4.0; 7.0)	7.0 (5.0; 7.0)	2.0 (2.0; 2.0)
26–35 years	20.2 ± 1.2	25.0 (18.0; 27.5)	6.5 (5.8; 7.0)	7.0 (6.0; 7.0)	2.0 (2.0; 2.0)
36+ years	19.5 ± 1.92	20.5 (17.2; 26.0)	5.0 (5.0; 6.00)	6.0 (5.25; 7.0)	2.0 (2.0; 2.0)
Daily number of cigarettes smoked					
20 or <	19.6 ± 1.9	22.5 (18.0; 26.2)	6.0 (4.0; 7.0)	6.0 (5.75; 7.0)	2.0 (2.0; 2.0)
21 or >	20.0 ± 1.6	22.0 (18.0; 27.0)	6.0 (5.0; 7.0)	7.0 (6.0; 7.0)	2.0 (2.0; 2.0)
Quit attempts					
< 5 times	19.6 ± 1.89	22.0 (17.5; 26.0)	6.0 (5.0; 6.50)	6.0 (5.0; 7.0)	2.0 (2.0; 2.0)
6–10 times	20.0 ± 1.63	25.0 (20.0; 27.0)	6.0 (5.0; 7.0)	7.0 (6.0; 7.0)	2.0 (2.0; 2.0)
11+ times	20.1 ± 1.71	23.0 (20.5; 27.5)	7.0 (5.0; 7.0)	7.0 (6.0; 7.0)	2.0 (2.0; 2.0)

*Statistically significant differences (at *p* < 0.05) were found between gender and craving reduction (*p* = 0.023); ratings of vaping satisfaction and age participants began smoking (overall *p* = 0.013); ratings of enjoyment and age began smoking (overall *p* = 0.017); and ratings of enjoyment and years smoked (overall *p* = 0.038)

**Post hoc tests indicated that the 16–17 year-old group had significantly lower Satisfaction (*p* = 0.015) and Enjoyment (*p* = 0.047) scores compared to the 13-year-old and younger group. The 16–17-year-old group also had significantly lower Enjoyment scores compared to the 14–15 year-old group (*p* = 0.026). There was a trend for higher Enjoyment scores between those who have been smoking 26–35 years compared to those smoking 36 years or more (*p* = 0.061 after adjusting for multiple comparisons)

The majority (96% [95% CI 89%, 97%]) of participants reported moderate to extreme reductions in nicotine craving with e-cigarette use as reflected in responses to the single item mCEQ question, “Does your e-cigarette immediately reduce your cravings for nicotine?” [34] Women scored significantly higher on the Craving Reduction domain compared to men (Median of 7.0 [6.00; 7.00] vs 6.0 [5.00; 7.00], *p* = 0.023). As seen in Table 4, there were no statistically significant associations observed between mCEQ domains and baseline age, type of residence, number of cigarettes smoked daily and number of quit attempts.

### The smoking cue appeal survey (SCAS)

The experience of smoking cues with e-cigarette use was evaluated using the SCAS. As can be seen in Table 6, almost all participants (99% [95% CI 95%, 100%]) reported a pleasant experience using their e-cigarettes. Pleasantness was especially attributed to a variety of sensory cues such as tasting, smelling and seeing the vapor and touching the device. As can be seen in Table 7, the age of participants was significantly related to the extent to which they experienced sensory cues associated with e-cigarettes as pleasant (*p* = 0.01). A Tukey HSD post hoc test showed that 18–34 years had a lower mean SCAS total score (− 14.55 ± 5.45) indicating more perceived pleasantness compared to 45+ years olds (− 11.54 ± 6.11), *p* = 0.012.

**Table 6:** Smoking Cue Appeal Survey (SCAS): Sensory Cues attributed to “Pleasantness”

Cue^a^	Proportion reporting pleasantness (%)	95% CI
Tasting the Vapor	98	94%, 99%
Smelling the vapor	88	82%, 93%
Touching the Device	78	71%, 84%
Seeing the Vapor	76	68%, 72%
Overall experience	99	95%, 100%
Smell of another’s use	85	78%, 90%
Taste of another’s use	35	28%, 44%
Sight of another’s use	50	42%, 58%

*Cue = the proportion of respondents who indicated use of their e-cigarette was pleasant, very pleasant, or extremely pleasant. NOTE: Questions on the SCASwere adapted to refer to e-cigarettes and e-cigarette use.

**Table 7:** Association of demographics and smoking history and total SCAS scores

	Total SCAS score mean ± SD	Overall *p value and **Post Hoc
Gender		
Male	− 12.82 ± 4.89	*p* = 0.506
Female	− 13.43 ± 6.31	
Baseline age		
18–34	− 14.55 ± 5.45	*p = 0.01
35–44	− 14.00 ± 4.61	**p = 0.012
45+	− 11.54 ± 6.11	
Residence type		
Rural	2.00 (2.00; 2.00)	*p* = 0.470
Suburban	2.00 (2.00; 2.00)	
Urban	2.00 (2.00; 2.00)	
Age began smoking		
< 13 years	− 14.00 (− 17.00; − 10.00)	0.384
14–15 years	− 14.00 (− 19.00; − 9.00)	
16–17 years	− 12.00 (− 15.00; − 9.00)	
18+ years	− 13.00 (− 18.00; − 7.00)	
Years smoked		
≤ 15 years	− 14.33 ± 5.24	0.070
16−25 years	− 14.05 ± 5.54	
26–35 years	− 13.32 ± 5.78	
36+ years	− 10.83 ± 5.06	
Daily number of cigarettes smoked		
20 or <	− 13.34 ± 5.35	0.859
21 or >	− 13.18 ± 5.68	
Quit attempts		
< 5 times	− 13.01 ± 5.23	0.764
6–10 times	− 13.79 ± 5.78	
11+ times	− 12.96 ± 5.73	

*ANOVA: *p* < 0.05.

**Post hoc tests indicated that those 18–34 years had a lower mean SCAS total score (− 14.55 ± 5.45) indicating more perceived pleasantness compared to 45+ years olds (− 11.54 ± 6.11), *p* = 0.012.

There were no statistically significant associations observed between SCAS scores and gender, age of initiation of smoking, years of smoking, type of residence, number of cigarettes smoked daily, and number of quit attempts.

### Discussion

The goal of this study was to characterize the extent and quality of respiratory sensations and sensory-related smoking cues associated with e-cigarettes. Further, we examined the extent to which participants believed sensory experiences accompanying e-cigarette use contributed to their cessation outcomes.

 In our study, all our participants substituted e-cigarettes for CTC after trying and failing to quit CTC using a FDA approved cessation medication. Evidence suggests that substituting an e-cigarette for CTC may improve cessation outcomes, and in some cases substitution may yield a more robust cessation outcome compared to approved cessation medications. Hajeck and colleagues [37] compared 1 year abstinence rates (*N* = 886) among an e-cigarette group and a nicotine replacement group and reported 18.0% and 9.9% rates of cessation, respectively. Six month CO-verified continuous abstinence rates combining a nicotine containing e-cigarette with a nicotine patch (7%, *n* = 35) was superior to a non-nicotine containing e-cigarette and nicotine patch (4%, *n* = 20) group, and a nicotine patch alone (2%, *n* = 3) [38]. Several comprehensive systematic reviews concluded that e-cigarettes may be effective substitutes to help smokers quit or reduce smoking, yet these findings are inconclusive due to the low quality of the studies or small sample sizes [39–41].

The majority of participants in our study rated the sensations from the vapor as important and attributed the sensory aspect of e-cigarette use to their success at quitting CTC. These findings suggest the possibility that sensory experiences may be missing links in the effectiveness of current cessation medications. In contrast to the sensory stimulation with e-cigarette use, approved cessation medications focus exclusively on interrupting the nicotine addiction. Except for the nicotine inhaler, which mimics some of the hand-to-mouth sensory-motor cues of smoking [42], and the nasal spray that may induce some of the respiratory sensations of smoking, none of these medications are designed specifically to replicate the sensory experiences of smoking. Further, mouth, throat, and nose irritations are reported by users of the inhaler and spray [43].

E-cigarettes are designed to simulate the smoking experience, similar to the cigarette substitutes introduced by Rose and others [15, 16]. Aerosol from a nicotine containing solution is inhaled, and many e-cigarettes have the same features as a traditional cigarette such as shape, a filter, and red or orange glowing tip [44] and are associated with smoking-specific cues such as visible smoke, which is actually vapor [25]. Better understanding e-cigarette users’ sensory experiences is vital to considering novel approaches or improving existing cessation interventions.

Regarding the sensory experiences of e-cigarette users in our study, participants endorsed satisfying and enjoyable sensations throughout the respiratory tract (i.e., throat, tongue, windpipe, nose and lungs). These findings are consistent with other research. In Dawkins and colleagues’ online survey of e-cigarette users [23], participants described sensations accompanying e-cigarette use as “satisfying.” In Berg and colleagues’ online survey of e-cigarette users [24], participants said the sensation of blowing out vapor and the flavors reinforced cessation beyond nicotine replacement alone. In our study, the greatest enjoyment was experienced on the tongue and in the throat. This finding is consistent with other research. In Etter and colleagues’ study [26], participants attributed smoking cessation success to the extent of “throat hit” experienced while using their e-cigarettes. In a study conducted by Barbeau and colleagues [25] e-cigarette using participants in focus groups also reported enjoying the feeling of “throat hit.”

Enjoyment varied by age of initiation of smoking. We found that those who began smoking at a younger age had greater enjoyment and satisfaction from e-cigarettes. There was a statistically significant difference between those who began smoking between 16–17 years of age and both those who began smoking at 14–15 years old and those who began smoking at 13 years of age or younger. In our sample, enjoyment was significantly related to smoking tenure. After adjusting for multiple comparisons, those who smoked 26–35 years tended to have more e-cigarette enjoyment than those who had smoked 36 or more years (p value of 0.061 for the pairwise comparison), suggesting that the test might be underpowered to detect the difference. The reason less established smokers may experience more enjoyment is not addressed specifically in the literature. It may be that it is more difficult for entrenched smokers to find enjoyment using the alternative e-cigarette because the enjoyment experienced during smoking has been firmly established. In fact, in a survey of cigarette smokers, the main reason for not transitioning to an e-cigarette included enjoyment of traditional cigarette smoking [45].

Enjoyment and satisfaction may influence use of e-cigarettes and CTC [45–49] therefore both are critical to consider. In fact, among a sample of college students between the ages of 18 and 23 years, enjoyment surpassed all reasons for daily and non-daily e-cigarette use, including quitting or reducing smoking and decreasing craving [47]. In a nationally representative sample of adolescents and young adults in the United States, Barker and colleagues found a robust association between enjoyment of e-cigarette use and intention to use e-cigarettes. According to the findings from the 2016 ITC Four Country Smoking and Vaping wave 1 (4CV1) surveys conducted in the United States, England, Canada, and Australia [50], the greatest motivation for e-cigarette use among ex-cigarette smokers was enjoyment. Therefore, understanding more about how length of smoking tenure and age of smoking initiation may impact enjoyment and satisfaction of e-cigarettes is important to address in future research.

Varied enjoyment/satisfaction may be explained by differences in the nicotine strength of users’ original CTC compared to the strength of the e-cigarette, participants’ levels of nicotine dependence, types of devices, and flavors. In our study, enjoyment and satisfaction were not correlated with number of cigarettes smoked per day or number of quit attempts, both associated with greater nicotine dependence [1, 51]. Further, there was no significant difference in enjoyment of e-cigarettes between genders. This could be related to the development of newer generation devices, increased product acceptability and accessibility, as compared to earlier research where males scored higher than females on attributes of enjoyment to maintain e-cigarette use [52], while using first (10%) and second (61%) generation devices.

Perhaps enjoyment may influence e-cigarette use differently based on gender. It is well established that gender differences exist with cigarette smoking behavior [52] and product choice [53]. However, research focused on gender influences of e-cigarette use behavior are still being established and is an area in need of further research [52].

Participants endorsed sensory cues accompanying e-cigarette use (e.g. tasting, smelling, seeing the vapor and touching the device). “Pleasantness” was attributed to these cues. Similar findings from a qualitative study attributed “biobehavioral feedback” to the efficacy of an e-cigarette for cessation. Participants in this study emphasized the sensory cues of e-cigarette use, such as seeing the vapor cloud when they exhaled as influencers for cessation [25]. In our study, 18–34-year-olds experienced greater “pleasantness” compared to those 45 or more years.

Moderate to extreme reductions in nicotine craving were reported by the majority (94%) of our sample.

Craving reductions with e-cigarette use are documented in the literature. A randomized controlled trial demonstrated a reduction in cigarette craving that was of the same magnitude as when a cigarette was smoked when using an e-cigarette after 4 h of abstinence from smoking [33]. Craving reductions have been documented in other studies [23, 54, 55]. Findings of a Web-based survey of 1672 e-cigarette users demonstrated an association between higher levels of nicotine in the e-cigarette, stronger “throat hit,” increased relief of cravings, smoking cessation, and “satisfaction” when using the e-cigarette [26]. Women experienced significantly greater craving reductions in our study. This is consistent with findings from an on-line survey of e-cigarette users in which females were more likely to agree that the e-cigarette dramatically reduced cravings for nicotine [23]. Expectations may influence gender differences in experiences with e-cigarette use. In an online survey, women were significantly more likely to maintain e-cigarette use for the purpose of dealing with stress or to control moods [56].

## Conclusions

In conclusion, the results of our study suggest the importance of sensory experiences when switching from CTC to a cessation aide. In our sample, sensory experiences influenced cessation outcomes. This finding underscores the importance of considering the sensory impact of smoking when designing interventions for cessation. It is conceivable that the absence of sensory stimulation may be a factor in the limitations of approved cessation medications to achieve robust and sustained cessation outcomes.

Our findings do not specifically support an e-cigarette as a substitute for CTC. The safety profile of e-cigarettes is still being evaluated and there exists a global debate about the appropriateness of promoting e-cigarettes as tools to help smokers quit. Rather, we urge fellow researchers and clinicians reflect on the seminal works of Cain [15, 17]. It is best to think seriously about the precision to which TI scientists design cigarettes to ingrain smoking behavior by engineering the perfect sensory influencing and nicotine supplying device. Is it possible that our cessation failure outcomes are still estimated to be as high as 50% 1-year postquit [8–12], because our approved cessation medications address only the nicotine addiction but not the sensory impacts of smoking? How would the lives of 1.1 billion smokers worldwide [13] be improved if our cessation interventions matched the design of the CTC by addressing both the sensory impact of smoking and the nicotine addiction? Current e-cigarettes already allow consumers to tailor their experience right down to an individual level, and so it is perhaps the concept of 'recognising and permitting pleasure' as an adjunct to nicotine replacement which must be adopted in the first place in order for the intervention to succeed.

At present, to our knowledge, e-cigarettes are the most viable CTC substitutes that provide the sensory experience and cues that appear to be important for smoking cessation. It is for this reason that we encourage further research of the e-cigarette for safety, substitution, and sensory impact. Further, it will be important for future research to determine which other types of devices might be helpful to create sensory cues and experiences that might help smokers quit.

Limitations should be considered when interpreting our results. Survey responses from our small non-representative sample may be biased based on affiliation. Participants were recruited through CASAA, an advocacy group for electronic nicotine devices. Further, the sample size was limited to 156 based on funding. Additionally, the sample excludes those who are less likely to utilize social media, electronics or the web. A standard 6-month cessation criterion, [57] rather than a 3-month cessation criterion for eligibility would enhance ability to compare results with other studies. No data were collected on the frequency of e-cigarette use, which may influence CTC cessation outcomes [58]. Nor were data collected regarding e-cigarette brands, nicotine content, or flavors, all of which have the potential to influence sensory experiences. In fact, by the year 2014, there were 466 e-cigarette brands and 7764 unique flavors of e-cigarettes identified by an internet search [59, 60]. A review of 16 brands of e-cigarettes found variations in levels of nicotine in the vapor from 0.5 to 15.4 mg [49].

Implications for future research include comparing sensory experiences and cessation outcomes among stratified samples of e-cigarette users of varying nicotine strengths, brands, and flavors, and including patterns of e-cigarette use as variables of interest. Adding a control group of “dual users” of both CTCs and e-cigarettes versus exclusive e-cigarette users would be of interest to determine if responses to the questionnaire are different between exclusive e-cigarette users (successful sustained abstinence from tobacco cigarettes) versus dual (quit failures) users.

In spite of these limitations, study findings add valuable insights. While the sensory influences of smoking behavior are well understood by current and seminal research, none of the approved cessation medications are designed with the sensory reinforcers of smoking behavior in mind. Our results suggest the potential importance of including such sensory influences in CTC cessation interventions.

## Data Availability

The datasets used and/or analyzed during the current study are available from the corresponding author on reasonable request.

## Abbreviations

CTC: Combustible tobacco cigarette
mCEQ: Modified Cigarette Evaluation Questionnaire
SCAS: Smoking Cue Appeal Survey
SD: Standard deviation
IQR: Interquartile range
ANOVA: Analysis of variance

## References

[1] Fagerström K (2012). Determinants of tobacco use and renaming the FTND to the Fagerström test for cigarette dependence. Nicotine Tob Res.

[2] Fiore MC, Jaén CR, Baker TB, Bailey WC, Benowitz N, Curry SJ, Wewers ME (2009). Treating tobacco use and dependence: 2008 update. Quick reference guide for clinicians.

[3] Pritchard WS, Robinson JH, Guy TD, Davis RA, Stiles MF (1996). Assessing the sensory role of nicotine in cigarette smoking. Psychopharmacology.

[4] Carpenter Carrie M, Wayne Geoffrey F, Connolly GN (2005). Designing cigarettes for women: new findings from the tobacco industry documents. Addiction.

[5] Cook Benjamin L, Wayne Geoffrey F, Keithly L, Connolly G (2003). One size does not fit all: how the tobacco industry has altered cigarette design to target consumer groups with specific psychological and psychosocial needs. Addiction.

[6] Behm FM, Schur C, Levin ED, Tashkin DP, Rose JE (1993). Clinical evaluation of a citric acid inhaler for smoking cessation. Drug Alcohol Depend.

[7] Rose JE, Behm FM, Westman EC, Johnson M (2000). Dissociating nicotine and nonnicotine components of cigarette smoking. Pharmacol Biochem Behav.

[8] Aubin T, Auger M, Genest M-H, Giroux G, Gornea R, Faust R, Leroy C, Lessard L, Martin J-P, Morlat T (2008). Discrimination of nuclear recoils from alpha particles with superheated liquids. N J Phys.

[9] Hughes JR, Stead LF, Lancaster T. Antidepressants for smoking cessation. Cochrane database of systematic reviews. In: The Cochrane library, Issue 06, 30.07.2011; 2011. https://cochrane.bvsalud.org/cochrane/main.php?lib=COC&searchExp=smoking&lang=p 10.1002/14651858.CD000031.pub2 15494986

[10] Stead LF, Perera R, Bullen C (2012). Nicotine replacement therapy for smoking cessation. Cochrane Database Syst Rev.

[11] West R, Baker CL, Cappelleri JC, Bushmakin AG (2008). Effect of varenicline and bupropion SR on craving, nicotine withdrawal symptoms, and rewarding effects of smoking during a quit attempt. Psychopharmacology.

[12] Gonzales D, Rennard SI, Nides M (2006). Varenicline, an α4β2 nicotinic acetylcholine receptor partial agonist, vs sustained-release bupropion and placebo for smoking cessation: a randomized controlled trial. JAMA.

[13] WHO. Tobacco. 2019. Retrieved 2 Feb 2020. https://www.who.int/news-room/fact-sheets/detail/tobacco.

[14] The health consequences of smoking—50 years of progress: a report of the surgeon general. Atlanta, GA: U.S. Department of Health and Human Services, Centers for Disease Control and Prevention, National Center for Chronic Disease Prevention and Health Promotion, Office on Smoking and Health; 2014.

[15] Cain WS, Gori GB, Bock FG (1980). Sensory attributes to cigarette smoking. Banbury report 3: a safe cigarette?.

[16] Rose JE (1988). The role of upper airway stimulation in smoking. Prog Clin Biol Res.

[17] Westman EC, Behm FM, Rose JE (1996). Dissociating the nicotine and airway sensory effects of smoking. Pharmacol Biochem Behav.

[18] Rose JE, Behm FM (1994). Inhalation of vapor from black pepper extract reduces smoking withdrawal symptoms. Drug Alcohol Depend.

[19] Rose JE, Tashkin DP, Ertle A, Zinser MC, Lafer R (1985). Sensory blockade of smoking satisfaction. Pharmacol Biochem Behav.

[20] Carpenter CM, Wayne GF, Connolly GN (2007). The role of sensory perception in the development and targeting of tobacco products. Addiction.

[21] Megerdichian CL, Rees VW, Wayne GF, Connolly GN (2007). Internal tobacco industry research on olfactory and trigeminal nerve response to nicotine and other smoke components. Nicotine Tob Res.

[22] US Food and Drug Administration. Vaporizers, E-Cigarettes, and other Electronic Nicotine Delivery Systems (ENDS). https://www.fda.gov/TobaccoProducts/Labeling/ProductsIngredientsComponents/ucm456610.htm. Accessed 18 Feb 2018.

[23] Dawkins L, Turner J, Roberts A, Soar K (2013). ‘Vaping’ profiles and preferences: an online survey of electronic cigarette users. Addiction.

[24] Berg CJ, Barr DB, Stratton E, Escoffery C, Kegler M (2014). Attitudes toward E-cigarettes, reasons for initiating E-cigarette use, and changes in smoking behavior after initiation: a pilot longitudinal study of regular cigarette smokers. Open J Prev Med.

[25] Barbeau AM, Burda J, Siegel M (2013). Perceived efficacy of e-cigarettes versus nicotine replacement therapy among successful e-cigarette users: a qualitative approach. Addict Sci Clin Pract.

[26] Etter J-F (2016). Throat hit in users of the electronic cigarette: an exploratory study. Psychol Addict Behav.

[27] Caponnetto P, Russo C, Bruno C, Alamo A, Amaradio M, Polosa R (2015). Electronic cigarette: a possible substitute for cigarette dependence. Monaldi Arch Chest Dis.

[28] Farsalinos KE, Romagna G, Tsiapras D, Kyrzopoulos S, Voudris V (2014). Characteristics, perceived side effects and benefits of electronic cigarette use: a worldwide survey of more than 19,000 consumers. Int J Environ Res Public Health.

[29] Gallus S, Lugo A, Pacifici R, Pichini S, Colombo P, Garattini S (2014). E-cigarette awareness, use, and harm perceptions in Italy: a national representative survey. Nicotine Tob Res.

[30] Dawkins L, Kimber C, Puwanesarasa Y, Soar K (2014). First- versus second-generation electronic cigarettes: predictors of choice and effects on urge to smoke and withdrawal symptoms. Addiction.

[31] Kistler CE, Crutchfield TM, Sutfin EL, Ranney LM, Berman ML, Zarkin GA (2017). Consumers’ preferences for electronic nicotine delivery system product features: a structured content analysis. Int J Environ Res Public Health.

[32] Soule EK, Maloney SF, Guy MC, Eissenberg T, Fagan P (2017). User identified positive outcome expectancies of electronic cigarette use: a concept mapping study. Psychol Addict Behav J Soc Psychol Addict Behav.

[33] Farsalinos KE, Polosa R (2014). Safety evaluation and risk assessment of electronic cigarettes as tobacco cigarette substitutes: a systematic review. Ther Adv Drug Saf.

[34] Cappelleri JC, Bushmakin AG, Baker CL, Merikle E, Olufade AO, Gilbert DG (2007). Confirmatory factor analyses and reliability of the modified cigarette evaluation questionnaire. Addict Behav.

[35] Murray HW, McHugh RK, Rowley TC, Sirota AD, Otto MW (2010). Development and validation of the smoking cue appeal survey. Subst Use Misuse.

[36] Nicholson AK, Borland R, Davey ME, Stevens M, Thomas DP (2015). Past quit attempts in a national sample of Aboriginal and Torres Strait Islander smokers. Med J Aust.

[37] Hajek P, Phillips-Waller A, Przulj D (2019). A randomized trial of e-cigarettes versus nicotine-replacement therapy. N Engl J Med.

[38] Walker N, Verbiest M, Kurdziel T (2019). Effectiveness and safety of nicotine patches combined with e-cigarettes (with and without nicotine) for smoking cessation: study protocol for a randomised controlled trial. BMJ Open.

[39] Hartmann-Boyce J, McRobbie H, Bullen C, Begh R, Stead LF, Hajek P (2016). Electronic cigarettes for smoking cessation. Cochrane Database Syst Rev.

[40] Malas M (2016). Electronic cigarettes for smoking cessation: a systematic review. Nicotine Tob Res.

[41] Rahman MA, Hann N, Wilson A, Mnatzaganian G, Worrall-Carter L (2015). E-cigarettes and smoking cessation: evidence from a systematic review and meta-analysis. PLoS ONE.

[42] Bergström M, Nordberg A, Lunell E, Antoni G, Långström B (1995). Regional deposition of inhaled 11C-nicotine vapor in the human airway as visualized by positron emission tomography. Clin Pharmacol Ther.

[43] Nides M (2008). Update on pharmacologic options for smoking cessation treatment. Am J Med.

[44] Orellana-Barrios MA, Payne D, Mulkey Z, Nugent K (2015). Electronic cigarettes—a narrative review for clinicians. Am J Med.

[45] Etter JF (2010). Electronic cigarettes: a survey of users. BMC Public Health.

[46] Majeed BA, Stanton CA, Dube SR, Sterling KL, Burns JD, Eriksen MP (2016). Electronic cigarette use among current smokers: a pilot qualitative study. Health Behav Policy Rev.

[47] Saddleson ML, Kozlowski LT, Giovino GA, Goniewicz ML, Mahoney MC, Homish GG (2016). Enjoyment and other reasons for electronic cigarette use: results from college students in New York. Addict Behav.

[48] Sussan TE, Shahzad FG, Tabassum E (2017). Electronic cigarette use behaviors and motivations among smokers and non-smokers [published correction appears in BMC Public Health. 2018 Jan 24;18(1):175]. BMC Public Health.

[49] Goniewicz ML, Kuma T, Gawron M, Knysak J, Kosmider L (2013). Nicotine levels in electronic cigarettes. Nicotine Tob Res.

[50] Yong HH, Borland R, Cummings KM (2019). Reasons for regular vaping and for its discontinuation among smokers and recent ex-smokers: findings from the 2016 ITC Four Country Smoking and Vaping Survey. Addiction.

[51] John U, Meyer C, Hapke U, Rumpf H-J, Schumann A (2004). Nicotine dependence, quit attempts, and quitting among smokers in a regional population sample from a country with a high prevalence of tobacco smoking. Prev Med.

[52] Piñeiro B, Correa JB, Simmons VN, Harrell PT, Menzie NS, Unrod M (2016). Gender differences in use and expectancies of E-cigarettes: online survey results. Addict Behav.

[53] Westmaas J, Langsam K (2005). Unaided smoking cessation and predictors of failure to quit in a community sample: effects of gender. Addict Behav.

[54] Bullen C, McRobbie H, Thornley S, Glover M, Lin R, Laugesen M (2010). Effect of an electronic nicotine delivery device (e cigarette) on desire to smoke and withdrawal, user preferences and nicotine delivery: randomised cross-over trial. Tobacco Control.

[55] Eissenberg T (2010). Electronic nicotine delivery devices: ineffective nicotine delivery and craving suppression after acute administration. Tob Control.

[56] Piñeiro B, Correa JB, Simmons VN (2016). Gender differences in use and expectancies of e-cigarettes: online survey results. Addict Behav.

[57] West R, Hajek P, Stead L, Stapleton J (2005). Outcome criteria in smoking cessation trials: proposal for a common standard. Addiction.

[58] Polosa R, Caponnetto P, Morjaria JB, Papale G, Campagna D, Russo C (2011). Effect of an electronic nicotine delivery device (e-cigarette) on smoking reduction and cessation: a prospective 6-month pilot study. BMC Public Health.

[59] Zhu S-H, Sun JY, Bonnevie E, Cummins SE, Gamst A, Yin L (2014). Four hundred and sixty brands of e-cigarettes and counting: implications for product regulation. Tobacco Control..

[60] Ajzen I (1991). The theory of planned behavior. Org Behav Hum Decis Process.

